# Mechanism of the effect of glycosyltransferase GLT8D2 on fatty liver

**DOI:** 10.1186/s12944-015-0040-3

**Published:** 2015-05-08

**Authors:** Yutao Zhan, Fei Zhao, Ping Xie, Leping Zhong, Dongnian Li, Qujing Gai, Li Li, Hongshan Wei, Lingqiang Zhang, Wei An

**Affiliations:** Department of Gastroenterology, Beijing Tongren Hospital, Capital Medical University, No.1 Dongjiaominxiang, Dongcheng District, Beijing, 100730 China; State Key Laboratory of Proteomics, Beijing Proteome Research Center, Beijing Institute of Radiation Medicine, Beijing, 102206 China; Institutes of Infectious Disease, Beijing Ditan Hospital, Capital Medical University, Beijing, 100015 China; Department of Cell Biology, Municipal Laboratory for Liver Protection and Regulation of Regeneration, Capital Medical University, 10 You An Men Wai Xi Tou Tiao, Beijing, 100069 China

**Keywords:** Glycosyltransferase, GLT8D2, HepG2 cells, Fatty liver

## Abstract

**Background:**

Recent studies have shown that some glycosyltransferases are involved in the development of nonalcoholic fatty liver disease **(**NAFLD). The objective of this study was to explore the effect and mechanism of glycosyltransferase GLT8D2 on fatty liver.

**Methods:**

Rat model of NAFLD was established by induction with high-fat-diet. The GLT8D2 expression in rat liver was examined using immunohistochemistry. Oil Red O staining and triglyceride assay were used to measure the effect of abnormal GLT8D2 expression on lipid accumulation in HepG2 cells. The expression levels of lipid metabolism-related key molecules, namely sterol regulatory element-binding protein-1c (SREBP-1c), stearoyl-coA desaturase (SCD), carnitine palmitoyltransferase-1 (CPT1) and microsomal triglyceride transfer protein (MTP), in HepG2 cells with abnormal GLT8D2 expression were determined by western blot analyses.

**Results:**

The expression of GLT8D2 was higher in the liver of rats with NAFLD than in the control rats, and GLT8D2 was mainly located around lipid droplets in hepatocytes. GLT8D2 expression increased in steatosis HepG2 cells compared with that in normal HepG2 cells. GLT8D2 positively regulated lipid droplet accumulation and triglyceride content in HepG2 cells. Upregulation or knockdown of GLT8D2 had no effect on the expressions of SREBP-1c, SCD or CPT-1 proteins in HepG2 cells. However, GLT8D2 expression negatively regulated the expression of MTP protein in HepG2 cells.

**Conclusion:**

GLT8D2 participated in NAFLD pathogenesis possibly by negatively regulating MTP expression. Specific inhibition of GLT8D2 via an antagonistic strategy could provide a potential candidate approach for treatment of NAFLD.

## Introduction

Non-alcoholic fatty liver disease (NAFLD) is one of the most common types of liver disease in the world [1,2]. With an increase in obese population, the incidence of NAFLD is expected to increase worldwide. The prevalence of NAFLD in the US is expected to increase by 50% in 2030 [3]. Although previously being recognized as benign, it is now clear that hepatic steatosis can evolve to more severe liver damage [4], including non-alcoholic steatohepatitis (NASH), hepatic fibrosis/cirrhosis and hepatocellular carcinoma (HCC) [5-7]. Moreover, NAFLD can promote the development and progression of diseases in other organs [8]. Several studies have confirmed that NAFLD is associated with high prevalence of cardiovascular disease, and it is an independent risk factor for cardiovascular disease in type 2 diabetes [9,10]. NAFLD also significantly increases the risk of diabetes [11]. Currently, there is no effective treatment for NAFLD, therefore, it is essential to explore the possible mechanism of NAFLD.

Glycosyltransferases constitute a large group of enzymes that transfer one or multiple molecules of sugar to a wide range of acceptor molecules such as lipids, proteins, hormones, secondary metabolites, and oligosaccharides [12]. These enzymes play key roles in many fundamental biological processes underpinning human health and disease, such as cell signalling, cellular adhesion, carcinogenesis, and cell wall biosynthesis in human pathogens [13]. Recent studies have shown that some glycosyltransferases affect the development of fatty liver disease. Fu et al. have reported that T-synthase (a glycosyltransferase) gene deficiency causes fatty liver disease in mice [14]. Ihara et al. have found that the transgenic mice expressing N-acetylglucosaminyltransferase III developed readily a fatty liver [15]. Recently, we have cloned a new glycosyltransferase gene GLT8D2 [16]. In the present study, we examined the GLT8D2 expression in fatty liver of rat, and the effect of aberrant expression of GLT8D2 on the accumulation of triglyceride and the several key molecules related to the accumulation of triglyceride in HepG2 cells.

## Materials and methods

### Materials

Lipofectamine 2000 reagent was purchased from Life Technologies (Carlsbad, CA,USA). Staining reagent of Oil Red O was purchased from Sigma (St. Louis, MO, USA). The triglyceride assay kit was obtained from Bioassay Systems (Hayward, CA, USA). The anti-GLT8D2 antibody was purchased from Santa Cruz(Santa Cruz, CA, USA). The anti-SREBP1 (sterol regulatory element-binding protein-1c) antibody, anti-MTP (microsomal triglyceride transfer protein) antibody, anti-CPT-1 (carnitine palmitoyltransferase-1) antibody and anti-SCD antibody were from Abcam (Beijing, China). Antibodies against His and GAPDH (glyceraldehyde-3-phosphate dehydrogenase) were from Marine Biological Laboratory (Beijing, China). Chemiluminescence kit for signal detection of Western blot is the product of Thermo Fisher (Boston, MA, USA). All other reagents were of analytical grade.

### Animal care

Male Sprague-Dawley (SD) rats weighed 150-180 g were purchased from the Academy of Military Medical Sciences (Beijing, China). Rats were randomly divided into two groups: the model group and the control group (12 rats in each group). A NAFLD rat model was established by feeding the rats with the high-fat Lieber-DeCarli liquid diet (energy 71% from fat, 11% from carbohydrates and 18% from protein) for 7 weeks. SD rats in the control group were fed the standard Lieber-DeCarli diet (energy 35% from fat, 47% from carbohydrates and 18% from protein). The diets were purchased from HFK bioscience company (Beijing, China). All animal procedures were carried out in accordance with the Animal Handling Guidelines of Capital Medical University).

### Immunohistochemistry

SD rats were euthanized after 7 weeks, and the liver was excised, fixed in 10% buffered formalin and embedded in paraffin. Sections were prepared for immunohistochemical array. Unstained sections were deparaffinized and rehydrated. After distilled water rinses, endogenous peroxidase was inactivated with 3% hydrogen peroxide for 3 min. The sections were then treated with 10 mM sodium citrate buffer (pH6.0) for 20 min for microwave antigen retrieval. The slides were incubated with primary antibody (anti- GLT8D2) in a moist chamber for 30 min at 37°C. The antibody against GLT8D2 was used at a dilution of 1:1000. After rinsed in distilled water, the sections were incubated with secondary antibody (goat anti-mouse IgG) for 30 min at 37°C, and then incubated with S-A/HRP at 37°C for 30 min. Signal development was performed using 3,3-diaminobenzidin (DAB) as a chromogen. Sections were counterstained with hematoxylin and mounted in glycergel. The negative control group was treated with the same steps as described above, but the primary antibody was replaced by phosphate-buffered saline (PBS).

### Cell culture and treatments

HepG2 cells were purchased from ATCC (Manassas, VA, USA) and maintained at the Institute of Infectious Disease, Capital Medical University. The cells were cultured in the Dulbecco’s Modified Eagle’s Medium (DMEM) supplemented with 10% fetal calf serum (FCS) in a 5% CO2-humidified atmosphere at 37°C. HepG2 cells were seeded in 6-well plates. Induction of steatosis was achieved using a 100 μmol/L oleic acid (OA) solution dissolved in 0.01 mM NaOH. The duration of the treatment to induce steatosis was 72 h. At 24 h after cell seeding, HepG2 cells were used for transfection and RNA interference with or without the incubation of OA for 72 h.

### Transfection and RNA interference

The plasmid GLT8D2 was transfected into HepG2 cells with Lipofectamine 2000 according to the manufacturer’s instructions. According to our previous study [16], we selected the strongest inhibitory GLT8D2 shRNA in this research. The GLT8D2 shRNA (5′-TGCTGTCATGTTGGCAACAATCACACGTTTTGGCCACTGACTGA CGTGTGATTTGCCAACATGA-3′), and non-target control shRNA (5′-TGCTGAAATGTACTGCGCGTGGAGACGTTTTGGCCACTGACTG ACGTCTCCACGCAGTACATTT-3′) were synthesized by Life Technologies and transfected into HepG2 cells using Lipofectamine 2000. 72 h post transfection, the cells were collected for further analysis.

### Oil Red O staining

HepG2 cells were grown on a coverslip, washed with phosphate-buffered saline and then fixed with 10% formalin solution for 5 min at room temperature. After fixation, cells were washed gently with 60% isopropanol and stained with the working solution of 0.5 g Oil Red O in 60% isopropanol for 15 min. The stained hepatocytes were washed with distilled water several times to remove unincorporated dye. Then, the samples were counterstained with hematoxylin for 5 min. Results were examined using a light microscope.

### Intracellular triglyceride content assay

HepG2 cells were pre-incubated in 25-cm^2^ cell culture flasks for 18-24 h and then transfected with *his-GLT8D2* plasmid and *GLT8D2* shRNA, respectively. The cells were cultured in DMEM with or without OA. After 72 h of incubation, cells were collected and centrifuged at 1000 g for 5 min. Cell pellets were washed with PBS once, resuspended in 400 μL PBS buffer and transferred to a micro-smashing tube for ultrasonication. After ultrasonication, the concentration of cellular triglyceride was determined using an EnzyChrom™ triglyceride assay kit (Bioassay Systems, Hayward, CA, USA) and normalized with protein concentration according to the protocol provided by the manufacturer.

### Western blot analysis

Cells were lyzed in HEPES [N-(2-hydroxyethyl) piperazine-N’-2-ethanesulfonic acid] lysis buffer (20 mM HEPES, 50 mM NaCl, 0.5% Triton X-100, 1 mM NaF and 1 mM dithiothreitol). Protein from each sample was separated by 10% SDS-PAGE and electrotransferred to nitrocellulose filter membranes. The membranes were blocked with 5% BSA in TBS for 1 h at room temperature and incubated overnight at 4°C using the indicated primary antibodies, followed by detection with the related secondary antibody and the Super Signal chemiluminescence kit (Thermo Fisher).

### Statistical analysis

Data are expressed as mean ± SD. The significance of differences was determined by *t*-test using the SPSS 17.0 software (SPSS, Chicago, IL, USA). Difference with a p value of less than 0.05 was considered statistically significant.

## Results

### Inducement of NAFLD in rat model

**Figure 1:**
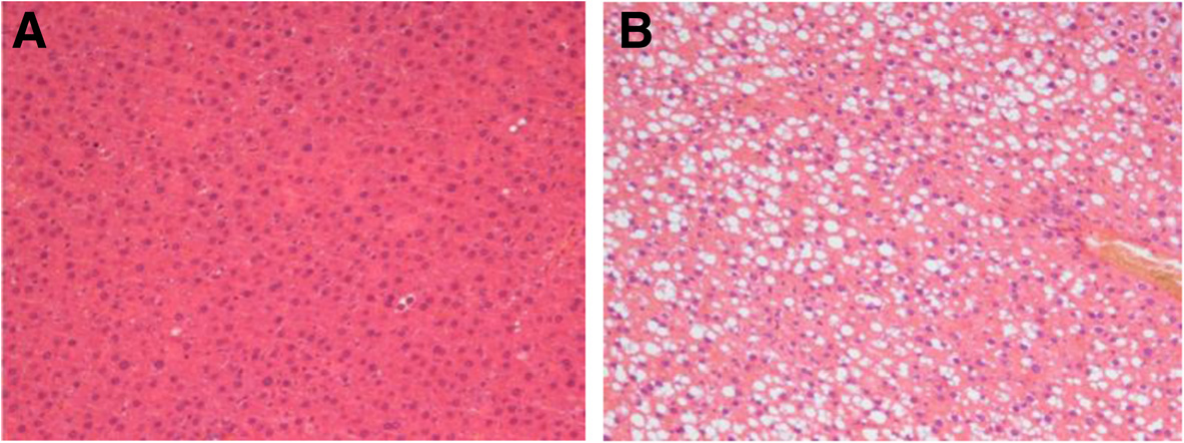
Successful inducement of rat NAFLD models. Specimens of rat liver were stained with HE (×200). **(A)** No obvious hepatocyte steatosis was found in control rat liver. **(B)** hepatocyte steatosis was obvious in NAFLD model rat liver.

No obvious steatosis was found in hepatocytes in rats in the control group. In contrast, there were a lot of steatotic hepatocytes in rats in the high-fat diet group (Figure 1). This suggested that the rat NAFLD model was established successfully.

### Expression of GLT8D2 in liver of rats with NAFLD

**Figure 2:**
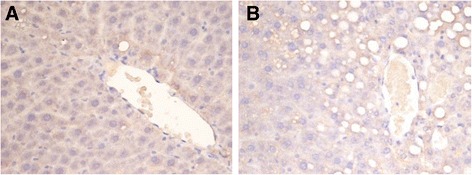
The expression of GLT8D2 in rat livers. GLT8D2 expression in rat livers was detected by immunohistochemistry (×400). **(A)** GLT8D2 expression was scattered in control rat liver. **(B)** GLT8D2 expression increased in NAFLD rat liver. GLT8D2 expression surrounded mainly the lipid droplets. Rat NAFLD model was induced by Lieber-DeCarli liquid diet for 7 weeks.

Immunohistochemical results of GLT8D2 in liver showed that the GLT8D2 expression was scattered in the rat liver of the control group. The expression of GLT8D2 was higher in the high-fat-diet animals than the control ones. In addition, GLT8D2 was obviously expressed surrounding the lipid droplets in the steatotic hepatocytes (Figure 2).

### Expression of GLT8D2 in HepG2 cells with steatosis

**Figure 3:**
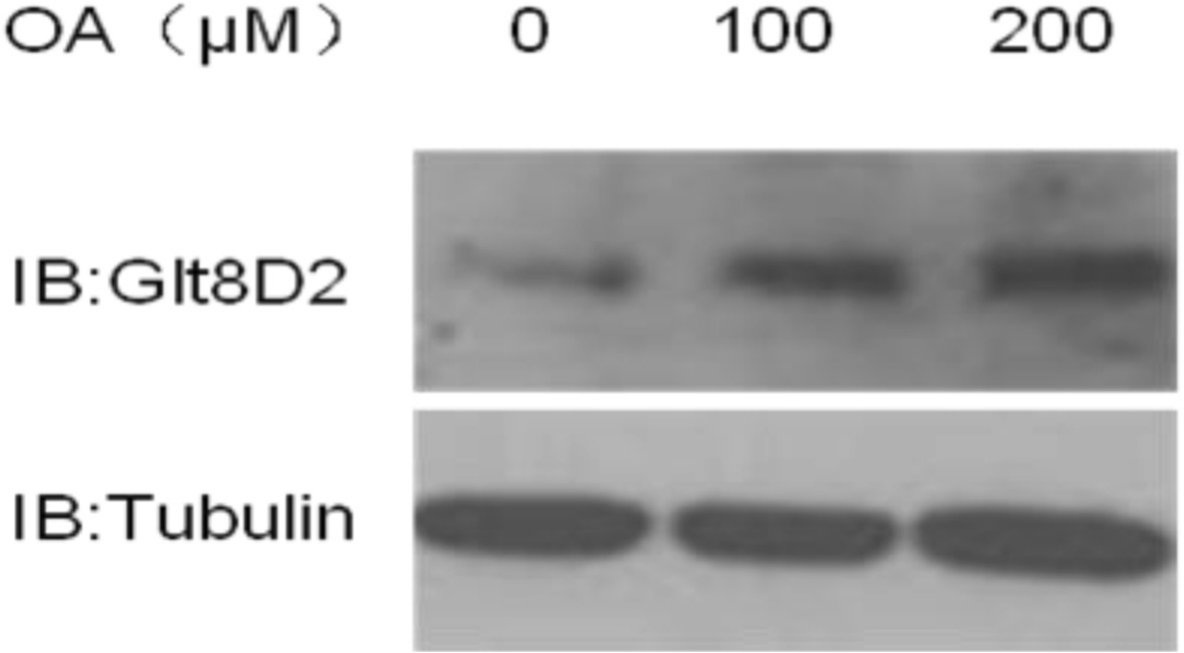
The effect of OA on GLT8D2 expression in HepG2 cells. HepG2 cells were treated with 0, 100 and 200 μM OA for 72 h, and then collected for western blot analysis. The GLT8D2 expression in HepG2 cells increased with the increase of OA concentration.

A cell model of steatosis *in vitro* was induced in HepG2 cells with OA at 100 μmol/L. After incubation for 72 h, OA treatment significantly increased the protein expression of GLT8D2 in HepG2 cells (Figure 3).

### GLT8D2 affected lipid accumulation in HepG2 cells

**Figure 4:**
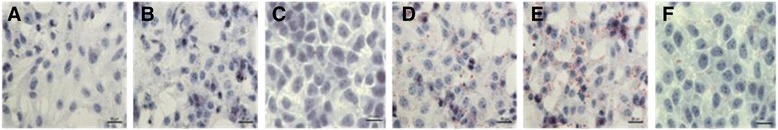
The effect of GLT8D2 abnormality on intracellular lipid droplet in HepG2 cells. Intracellular lipid droplet was tested by staining with Oil Red O. Enhanced GLT8D2 for 72 h increased lipid droplet accumulation in HepG2 cells with or without OA, especially in HepG2 cells with OA. Inhibitory GLT8D2 for 72 decreased lipid accumulation in HepG2 cells with OA. **(A)** HepG2 cells. **(B)** HepG2 cells with GLT8D2 transfection. **(C)** HepG2 cells GLT8D2 RNA interference. **(D)** HepG2 cells with OA. **(E)** HepG2 cells with OA and GLT8D2 transfection. **(F)** HepG2 cells with OA and GLT8D2 RNA interference.

**Figure 5:**
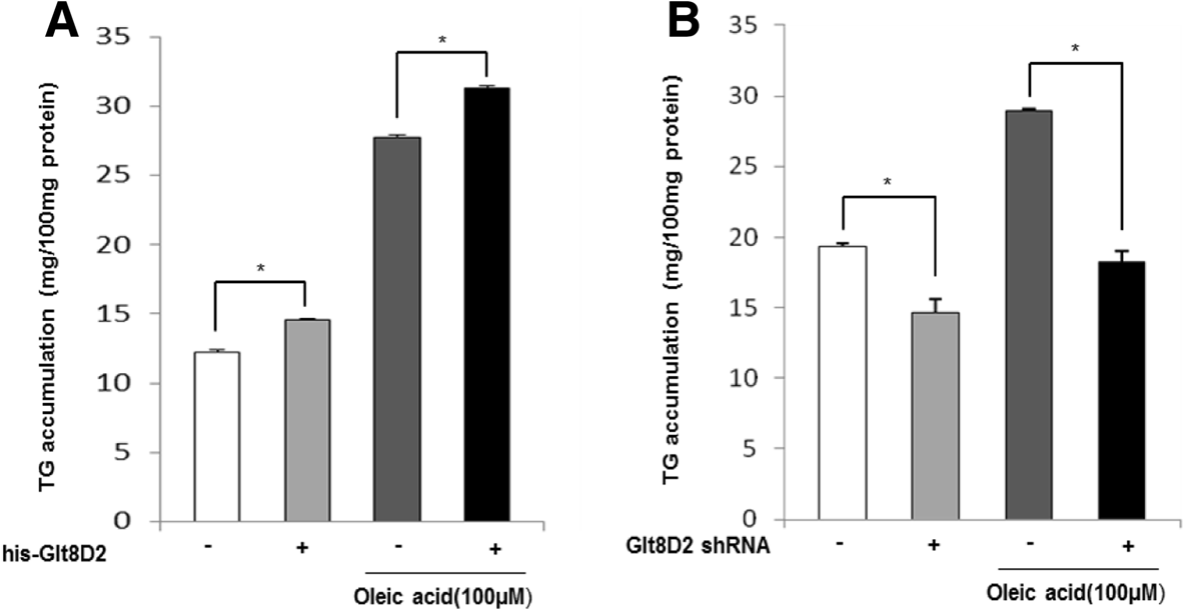
The effect of GLT8D2 abnormality on triglyceride content in HepG2 cells. **(A)** Enhanced GLT8D2 increased triglyceride content in HepG2 cells with or without OA. **(B)** Inhibitory GLT8D2 decreased triglyceride content in HepG2 cells with OA. Data are expressed as means ± SD (*n* = 6); * *p* < 0.05 compared with cells with normal GLT8D2, respectively.

In order to investigate whether GLT8D2 affected hepatocyte steatosis, HepG2 cells were transfected with his-*GLT8D2* plasmid or *GLT8D2* shRNA, respectively, and cultured continuously under non-fat-loading and fat-loading conditions. Lipid accumulation was examined after Oil Red O staining. As shown in Figure 4, under the non-fat-loading and fat-loading conditions, the overexpression of GLT8D2 correlated with an increase in the amount of lipid droplets in HepG2 cells. However, knockdown of *GLT8D2* by shRNA could reverse or alleviate the lipid droplet accumulation in hepatocytes as compared with *GLT8D*2 transfection. To quantify the effect of GLT8D2 on lipid accumulation, we next measured triglyceride concentration in cell lysate. In *GLT8D2*-overexpressing HepG2 cells, the levels of triglyceride in cells cultured under fat-loaded conditions was increased by 7.6% and 10.4%, respectively (Figure 5A). In contrast, the average intracellular triglyceride concentration in cells with GLT8D2 knockdown were reduced by 24.4% and 36.8% than those in the control cells under the fat-loading conditions, respectively (Figure 5B). These data suggested that GLT8D2 positively regulated lipid accumulation in HepG2 cells.

### Regulation of proteins involved in lipid homeostasis by GLT8D2

**Figure 6:**
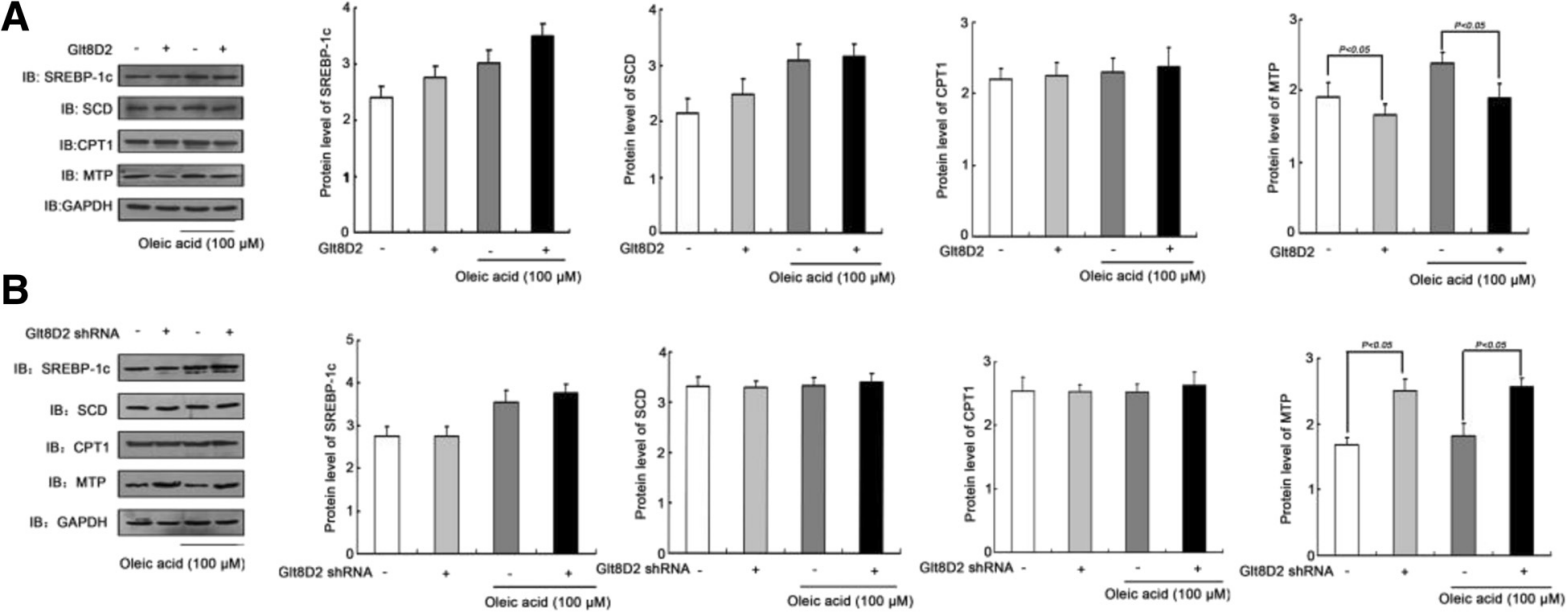
The effect of GLT8D2 abnormality on the expressions of lipid synthesis and metabolism-related molecules (SREBP-1c, SCD, MTP and CPT-1). **(A)** Enhanced GLT8D2 decreased MTP expression in HepG2 cells with or without OA. Enhanced GLT8D2 has not obvious affect on the SREBP-1c, SCD and CPT-1 expressions in HepG2 cells with or without OA. **(B)** Inhibitory GLT8D2 increased MTP expression in HepG2 cells with or without OA. Inhibitory GLT8D2 has not obvious affect on the SREBP-1c, SCD and CPT-1 expressions in HepG2 cells with or without OA. Data are expressed as mean ± SD (*n* = 6); * *p* < 0.05 compared with cells with normal GLT8D2, respectively. White square without GLT8D2 and without OA. Light gray square with GLT8D2 and without OA. Dark gray square without GLT8D2 and with OA. Black square with GLT8D2 and with OA.

To elucidate the mechanism of the effect of GLT8D2 on hepatocyte steatosis, we next examined the expressions of proteins involved in lipid homeostasis after GLT8D2 overexpression or knockdown (Figure 6). Microsomal triglyceride transfer protein (MTP) is an essential factor for very low density lipoproteins (VLDL) assembly and secretion. VLDL is the major lipoproteins responsible for transporting triglyceride from the liver to extra-hepatic tissues. Significant down-regulation of MTP in HepG2 cells was observed when GLT8D2 was overexpressed under both non-fat-loading and fat-loading conditions. Conversely, GLT8D2 knockdown increased MTP expression in HepG2 cells under both non-fat-loading and fat-loading conditions. Those results suggested that MTP could be a potential impaired molecule by GLT8D2 during intracellular lipid excretion. Other lipid synthesis and metabolism-related molecules, such as SREBP-1c, SCD proteins, and even CPT-11, one of the rate-limiting enzymes of the mitochondrial fatty acid β-oxidation pathway, seem not to be affected by GLT8D2.

## Discussion

Glycosyltransferases encompass 1% of all sequenced genomes [17]. There are over 65,000 glycosyltransferase sequences within the Carbohydrate-Active enzyme data base in 2011, comprising 89 families based on sequence similarity [13]. There are 105 glycosyltransferase structures in the Protein Data Bank [13]. To date, there 97 CAZy glycosyltransferase families (CAZy, http://www.cazy.org/GlycosylTransferases.html). As a member of the glycosyltransferase 8 family, the human GLT8D2 is a 349 amino acid single-pass type II membrane protein encoded by a gene located on chromosome 12q23.3. The first six amino acid residues extend to the cytoplasm, residues 7-24 are the transmembrane domain resided in the cell membrane; and residues 25-349 are in the luminal compartments [16]. Moylan et al. have reported that the *GLT8D2* gene is up-regulated in patients with severe NAFLD [1]. In the present study, we found that GLT8D2 expression increased in fatty liver of rats compared with normal liver, and that GLT8D2 was mainly expressed around lipid droplets. In the in vitro study, we also found that GLT8D2 expression increased in steatosis HepG2 cells compared with normal HepG2 cells. Further study showed that high GLT8D2 expression increases the accumulation of triglyceride in HepG2 cells. These data suggested that GLT8D2 might play an important role in the pathogenesis of NAFLD.

NAFLD is characterized by the excessive accumulation of triglyceride in hepatocytes [18]. Triglyceride is formed through the esterification of free fatty acids (FFAs) and glycerol. FFAs arise in the liver from three distinct sources [19]: (a) recirculation of non-esterified fatty acids from peripheral tissues (some from adipose tissues and some from skeletal muscle); (b) de novo lipogenesis (DNL) within hepatocytes and (c) dietary sources. Adipose tissue is the main source of liver FFAs. Approximately 60% of liver triglyceride is derived from FFA influx from the adipose tissue, 25% are from DNL, and 15% are from diet [20]. FFAs in liver have three major fates. They can be β-oxidized in mitochondria to produce energy and ketone bodies, re-esterified to triglyceride and stored in lipid droplets, or coupled to apolipoproteins and secreted as a constituent of VLDL [21]. Hence, hepatic fat accumulation can occur as a result of increased triglyceride synthesis, decreased FFAs oxidation and/or decreased fat export. DNL is controlled primarily at the transcriptional level [22,4]. Sterol regulatory element-binding protein-1c (SREBP-1c) is a major transcription factor that promotes the expression of lipogenic genes. It can activate all genes required for lipogenesis, such as SCD [23]. SCD is a key enzyme of fatty acid biosynthesis. Mitochondrial fatty acid β-oxidation is controlled by carnitine palmitoyltransferase-1 (CPT-1). CPT-1 is the rate-limiting enzyme of the mitochondrial fatty acid β-oxidation pathway [24,25]. Fatty acids are activated to form fatty acyl-CoAs. CPT-1 catalyzes the conversion of acyl-CoA to acyl-carnitine, which then enters into the mitochondria for fatty acid β-oxidation [26]. VLDL is the major lipoproteins transporting triglyceride from the liver to extrahepatic tissues [27]. It is assembled in endoplasmic reticulum in hepatic cells [28]. Each VLDL particle is stabilized by a single molecule of apolipoprotein B100 (apoB 100). ApoB 100 is a long polypeptide that is lipidated with triglycerides within the lumen of ER while it is being translated and translocated across the ER membrane.

MTP has both apoB 100 binding and lipid transfer domains [29]. MTP is an essential factor for VLDL assembly and secretion. To explore the effective molecules response to GLT8D2 in the accumulation of triglyceride in hepatocytes, we investigated the alternations of several key molecules related to the hepatic lipid accumulation as mentioned above. We found that the transfection of *GLT8D2* gene did not affect the expressions of SREBP-1c, SCD and CPT1, but actually decreased the MTP expression in HepG2 cells, and inhibitory GLT8D2 increased MTP expression in HepG2 cells. Combined with our previous results that GLT8D2 up-regulates the levels of apoB100 protein in HepG2 cells [16], we speculate that the inhibition of MTP expression by GLT8D2 may be the major mechanism resulting in accumulation of triglyceride in HepG2 cells. The detail mechanisms of GLT8D2 regulating MTP are still unknown thus need further study.

Based on these results, we conclude that the impairment of MTP function by GLT8D2 is attributed to the enhanced accumulation of triglyceride in hepatocytes during development of NAFLD, and specific inhibition of GLT8D2 via an antagonistic strategy could provide a potential candidate approach for treatment of NAFLD.

## Abbreviations

apoB 100: Apolipoprotein B100
CPT1: Carnitine palmitoyltransferase-1
DNL: De novo lipogenesis
FFAs: Free fatty acids
MTP: Microsomal triglyceride transfer protein
NAFLD: Non-alcoholic fatty liver disease
NASH: Non-alcoholic steatohepatitis
OA: Oleic acid
SCD: Stearoyl-coA desaturase
SREBP-1c: Sterol regulatory element-binding protein-1c
VLDL: Very low density lipoproteins
